# Evaluation of a point-of-care testing platform against a laboratory HbA1c measurement method in a South African tertiary-level diabetes clinic

**DOI:** 10.1016/j.plabm.2026.e00547

**Published:** 2026-07-01

**Authors:** Bibi Mohungoo Khan, Sean Currin, Farzahna Mohamed, Doreen Jacob

**Affiliations:** a Department of Chemical Pathology, Faculty of Health Sciences, University of the Witwatersrand, Johannesburg, South Africa; b National Health Laboratory Service, South Africa; c Department of Internal Medicine, Division of Endocrinology and Metabolism, Charlotte Maxeke Johannesburg Academic Hospital, Faculty of Health Sciences, University of the Witwatersrand, South Africa

**Keywords:** Diabetes, HbA1c, Point of care, Hb variants, HPLC, Immunoassay, Method comparison

## Abstract

**Background:**

Diabetes mellitus (DM) is a major global issue, with a prevalence of 11.1%. In Africa, South Africa is recognized as having one of the highest prevalences. Limited access to timely glycated haemoglobin (HbA1c) testing may delay treatment adjustments for diabetic patients. Point-of-care (POC) analysers provide faster results, potentially enhancing diabetes management. This study aimed to evaluate the performance of a POC device compared to a laboratory-based method, including the effect of potentially interfering haemoglobin (Hb) variants.

**Methods:**

HbA1c was measured in whole blood and capillary samples from 63 diabetic patients using the Finecare™ HbA1c POC device and compared to the Bio-Rad Variant II, a laboratory method utilising ion-exchange high-performance liquid chromatography (HPLC). 43 whole blood samples identified with Hb variants from routine laboratory HPLC analysis were also assessed on the POC device.

**Results:**

The POC device demonstrated a strong correlation with HPLC for both capillary (r = 0.94, 95% CI 0.90—0.96) and whole blood samples (r = 0.94, 95% CI 0.91—0.97). Mean bias was 1.3% (95% CI -1.2—3.8) for capillary vs. HPLC, and 4.5% (95% CI 2.1—6.9) for whole blood vs. HPLC. However, POC HbA1c in samples with Hb variants showed poor correlation with HPLC (r = 0.60, 95%CI 0.37-0.77) and a mean bias of −2.8% (95%CI -7.2—1.5).

**Conclusion:**

The Finecare™ HbA1c POC device performed acceptably for both capillary and whole blood samples, comparable to the HPLC method. We recommend capillary samples for routine monitoring but advise caution in populations with a high prevalence of Hb variants.

## Highlights

• Wondfo Finecare™ HbA1c POC demonstrated good agreement with HPLC method.
• Capillary samples demonstrated lower bias than whole blood samples on the POC device.
• POC performance was affected by presence of Hb variants, indicating possible assay interference.
• POC HbA1c suitable for HbA1c monitoring, not diagnosis, particularly in resource-limited settings.

## 1 Background

Diabetes mellitus (DM) is a major global health concern, affecting 589 million people worldwide with a prevalence of 11.1% [1]. In South Africa, DM ranks as the second leading cause of death and carries a prevalence of 7.2%, amongst the highest in Africa [2,3]. Early detection and effective management of DM, including addressing complications, are vital in reducing the associated morbidity and mortality. To tackle this challenge, the World Health Organization (WHO) has set ambitious global targets of ensuring that 80% of people with diabetes are diagnosed, and that 100% of those with type 1 DM have access to affordable insulin and blood glucose self-monitoring by 2030 [4,5].

Glycated Haemoglobin (HbA1c) measurement plays an important role in the diagnosis and monitoring of diabetic patients. It reflects the average blood glucose concentration over 2-3 months and is currently the most widely used index of average glycaemia for routine monitoring and prevention of DM complications [6]. The WHO and American Diabetes Association (ADA) endorse the use of HbA1c, and landmark studies such as the United Kingdom Prospective Diabetes Study (UKPDS) and Diabetes Control and Clinical Trials (DCCT) have established its correlation with diabetic complications [[7], [8], [9], [10]]. Currently HbA1c can be measured in laboratories by different methods, including immunoassay, enzymatic assay, capillary electrophoresis, boronate affinity and ion-exchange High Performance Liquid Chromatography (HPLC) [6]. HPLC coupled with mass spectrometry or capillary electrophoresis is considered the laboratory reference method [11].

All major guidelines, such as the ADA, the European Association for the Study of Diabetes (EASD) and the Society of Endocrinology, Metabolism and Diabetes of South Africa (SEMDSA) recommend individualised glycaemic targets in diabetic patients with regular HbA1c testing [[12], [13], [14]]. However, adherence to such guidelines is often suboptimal, especially in resource limited settings, such as South Africa, where delays in laboratory testing have been shown to impact clinical decision making [[15], [16], [17], [18]]. Point-of-care (POC) testing may offer a promising solution by utilising alternative sample types and providing immediate access to HbA1c results, without the need to access a centralised laboratory [19,20]. POC devices are only fit-for-purpose if they meet the necessary analytical performance specifications recommended for their clinical application. In the case of HbA1c, this should include the effect of potential interferents, which the ADA recognises as especially important for POC devices [21].

Haemoglobin (Hb) variants such as HbS, HbC, HbE, and HbD are known to interfere with certain HbA1c measurement techniques [[21], [22], [23]]. With an estimated global prevalence of around 7%, these Hb variants are usually asymptomatic in heterozygous carriers [24]. Their distribution is expanding due to global migration, a trend also reflected in South Africa, where prevalence varies between 0.2% and 1% depending on the ethnic group [25,26]. While certain laboratory methods can potentially detect and differentiate these variants, immunoassay-based methods, including POC devices, may not, which could lead to potential inaccuracies [21,23,27].

The aim of this study was to evaluate the analytical performance of the Wondfo® Finecare™ FIA Meter Plus FS-113 HbA1c Rapid Quantitative Test for HbA1c measurement, and to assess the potential effect of Hb variants on the POC device.

## 2 Materials and methods

### 2.1 Setting

This was a cross-sectional study conducted at the diabetic clinic at Charlotte Maxeke Johannesburg Academic Hospital (CMJAH) in Johannesburg, South Africa from February 2024 till May 2024. Laboratory samples were analysed at the National Health Laboratory Service (NHLS) laboratory at CMJAH. The study was approved by the Human Research Ethics Committee of the University of the Witwatersrand (WHREC Medical, reference M230908).

### 2.2 HbA1c measurement techniques

#### 2.2.1 POC method

The Wondfo ® Finecare ™ FIA Meter Plus FS- 113 HbA1c Rapid Quantitative Test (Guangzhou Wondfo Biotech Co., Ltd, P.R. China) is based on fluorescence immunoassay technology. Sample testing was carried out according to the manufacturer's instructions. The measuring range is 4.0-14.5%.

#### 2.2.2 Laboratory HPLC method

The Bio-Rad Variant ™ II Hemoglobin A_1_C Testing system (Bio-Rad Laboratories, Inc, California, USA) was used as the reference laboratory method. This HPLC system uses ion-exchange high performance liquid chromatography to quantify HbA1c. The program is certified by the National Glycohemoglobin Standardisation Program (NGSP) as having documented traceability to the DCCT reference method. The reportable range is 3.1-18.5%.

### 2.3 Study design

The study consisted of two independent cohorts.

- Cohort A: Known diabetic patients attending a diabetic clinic, used to compare capillary and venous HbA1c measurements on POC device with laboratory HPLC results.
- Cohort B: Whole blood remnant samples with Hb variants identified during routine laboratory HbA1c analysis and unrelated to Cohort A.

### 2.4 Sample collection and analysis

#### 2.4.1 HbA1c from Diabetic Clinic Patients (cohort A)

**Fig. 1:**
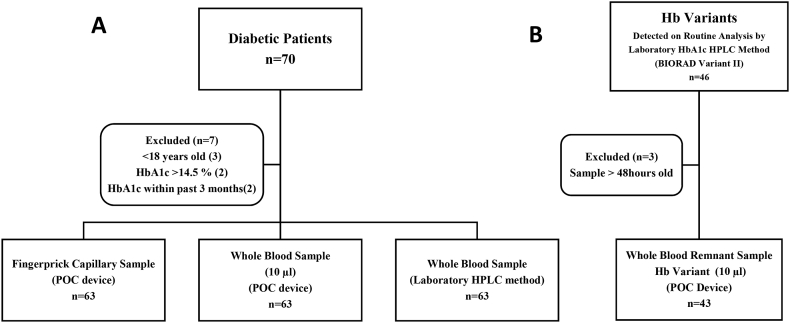
Sample collection from (A) Diabetic Clinic Patients (B) Hb Variants.

70 known diabetic patients attending the clinic for routine management were recruited by convenient sampling between 29 February 2024 and 18 April 2024, with eligibility criteria requiring participants to be ≥ 18 years old and no HbA1c testing within the preceding 3 months. All participants received a verbal explanation of the study and were provided with a patient information sheet. Written informed consent was obtained prior to participation. Ethnicity was determined through self-reporting. Patients were excluded if their laboratory HbA1c results fell outside the analytical range of the POC device (4-14.5%). After excluding 7 patients, 63 remained eligible for the study (Fig. 1 A).

After cleansing the puncture site with a 70% Isopropyl Alcohol swab and allowing the site to dry, a capillary fingerprick sample was obtained using a 21G disposable Mediject safety lancet, ensuring the finger was not “milked”. A capillary tube was used to collect the sample (10 μl), which was added to the pre-filled buffer tube provided by the manufacturer and mixed by shaking for more than 1 min 75 μL of the mixed sample was then pipetted into the sample well of the test cartridge and analysed on the POC device according to the manufacturer's instructions. Subsequently, venous blood was drawn by a trained phlebotomist into a BD Vacutainer ® EDTA tube, using BD Vacutainer ® Eclipse ™ 21G blood collection needles. From this whole blood sample, 10 μl of venous whole blood was pipetted out and added to the provided buffer. 75 μl of the mixed sample was pipetted out and immediately analysed on the POC device, while the remaining EDTA whole blood sample was stored at room temperature and analysed within 6 h using the HPLC method in the laboratory (Fig. 1A).

#### 2.4.2 Hb Variant samples (cohort B)

Samples with potential Hb variants were identified during routine analysis of HbA1c on the Bio-Rad Variant ™ II Hemoglobin A1C instrument between 8 February 2024 and 17 May 2024. This instrument has been shown to be effective at identifying potential Hb variants based on retention time and examination of chromatogram peaks [28]. The manufacturer has established that unless the combined area of Hb variant is ≥ 60% on the chromatogram, HbA1c can be reported [29]. Using this criteria, 46 samples with potential Hb variants were initially identified, which included HbS, and HbE,D. After excluding 3 samples which were beyond the 48-h stability period, 43 samples were eligible for the study, and no non reportable results due to interference were identified.

Whole blood remnant samples (10 μl) were pipetted out of the EDTA tube and mixed into the provided buffer and 75 μl of the mixed sample was analysed on the POC device within its stability period (Fig. 1B).

### 2.5 Precision verification study

A precision verification study was performed on the Finecare™ POC device according to the CLSI EP15-A3 guidelines [30]. Third-party quality control (QC) material (Bio-Rad Lyphochek Diabetes Control from Bio-Rad Laboratories Inc.) was analysed five times per day over five consecutive days, using two QC levels. The results of the precision study were compared to the manufacturer's claim as well as the recommended International Federation of Clinical Chemistry and Laboratory Medicine (IFCC) analytical performance specifications (see Section 2.7).

### 2.6 Method comparison study

A method comparison study was performed for each of the two study cohorts according to the CLSI EP 09-A3 guidelines [31].

In the diabetic clinic patient cohort (Cohort A), capillary fingerprick HbA1c results from the POC device were compared to the laboratory HPLC method. Venous whole blood HbA1c results from the POC device were also compared to the HPLC method. Additionally, POC capillary HbA1c results were directly compared to POC whole blood results.

In the Hb variant samples cohort (Cohort B), laboratory HPLC results were compared to POC whole blood remnant results.

### 2.7 Statistical analysis

Microsoft Excel® for Microsoft 365 (Version 2507) was used for the precision verification study, and MedCalc ® (MedCalcSoftware Ltd, Copyright © 1993-2024, Version 23.0.2) was used for statistical analysis of the method comparison study.

For the precision verification study, within-assay precision and within-laboratory precision for each QC level was assessed by calculating the mean and coefficient of variation (CV) using the ANOVA method, as described in CLSI EP 15- A3.

In the method comparison study, outliers were identified using the Tukey method, and normality of data distribution was assessed using the Shapiro-Wilk test. Deming regression with Pearson's correlation coefficient was used for the diabetic patient cohort and Passing-Bablok regression with Spearman's rank correlation coefficient, was used for the Hb Variants samples due to the presence of outliers. Bland Altman analysis for each cohort was used to assess the difference between methods and sample types, with %mean difference (not % HbA1C NGSP unit) used to represent the difference. The laboratory HPLC method was considered as the reference method when the two methods were compared to each other and mean of POC capillary and POC whole blood as the reference when the two sample types were compared to each other.

In terms of Analytical Performance Specifications, the allowable CV and Bias were calculated from the Total Allowable Error (TE_a_) based on the commonly used equation TE_a_ = 2(CV) + Bias [32]. The allowable TEa for HbA1c measurement was set at 6.9%, based on IFCC recommendations [33,34]. Therefore, the allowable bias was derived as ±3.5%, and the derived allowable imprecision or CV was 1.7%. Results from the study were evaluated against these criteria and the manufacturer's claims (CV<10%) to determine whether the POC device met the expected analytical performance specifications.

## 3 Results

### 3.1 Precision verification study results

Within-assay and within-laboratory CVs were within the manufacturer's stated limits (<10%) but exceeded the IFCC-derived CV target of 1.7%. The QC replicate results showed small biases between the device mean and the assigned mean values at both levels, which were within the derived allowable bias of 3.5%. Total error (TE) (derived from TEa = 2(CV) + bias), exceeded the IFCC target of 6.9% for Level 1 only. Precision verification results are summarised in Supplementary Table 1.

### 3.2 Study population (method comparison) results

Demographics and sample characteristics are summarised in Table 1, Table 2.

**Table 1:** Characteristics of diabetic clinic patients.

**n = 63**	
**Age, (Range in years)**	53 (18-79)
**Ethnicity, n (%)∗**	
Black	50 (79.4%)
White	9 (14.3%)
Indian	3 (4.8%)
Coloured	1 (1.6%)
**Sex, n (%)**	
Female	37 (58.7%)
Male	26 (41.3%)
**Laboratory HbA1c (by HPLC method), %**^a^	8.2 (6.95-10.35)
**POC Capillary HbA1c, %**^a^	8.1 (6.65-9.85)
**POC Whole Blood HbA1c, %**^a^	7.8 (6.25 -10)

Data presented as median (interquartile range, IQR) or n (percentage, %) as appropriate. HbA1c, glycated hemoglobin; POC, Point-of-care.

a HbA1c is NGSP (%) units.

**Table 2:** Characteristics of Hb variant samples.

**n = 43**	
**Hb Variant Type, n (%)**	
Hb S	24 (55.8%)
Hb E,D	19 (44.2%)
**Laboratory HbA1c (by HPLC method), (%)**^a^	6.8 ± 1.6
**POC Whole Blood HbA1c, (%)**^a^	7.0 ± 2.0

Data presented as mean ± standard deviation or n (percentage, %) as appropriate. HbA1c, glycated hemoglobin; HPLC, High-Performance-Liquid-Chromatography; POC, Point-of-care.

a HbA1c is NGSP (%) units.

#### 3.2.1 Diabetic Clinic Patients results

**Fig. 2:**
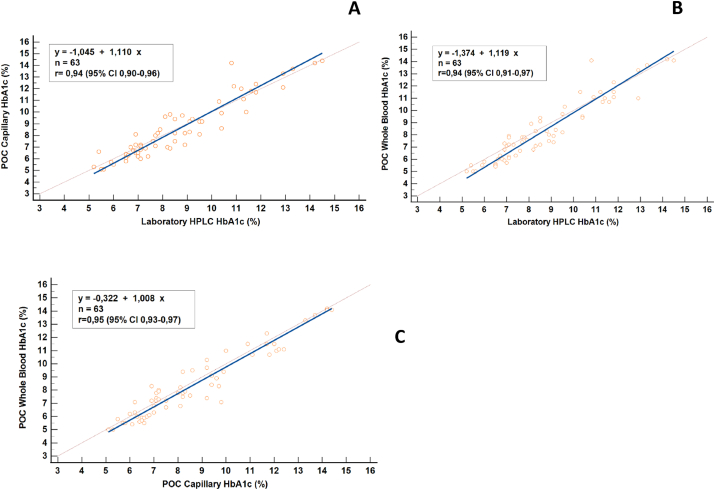
Method correlation of the BIORAD Variant II HPLC method compared with Finecare™ POC capillary samples (A), with Finecare™ POC whole blood samples (B) and Finecare™ Capillary Sample compared to Whole Blood sample (C) using Deming Regression Analysis. The dashed purple line represents the line of identity (y = x), the solid blue line is the Deming regression line.

As illustrated in Fig. 2, there was strong agreement between the different measurement methods and different sample types. For HPLC vs POC capillary samples (Fig. 2A), Deming regression yielded a slope of 1.11 (95% CI: 0.99 — 1.22) and a y-intercept of −1.05 (95% CI: −1.93 to −0.16), with a Pearson correlation coefficient of r = 0.94 (95% CI: 0.90 — 0.96). A similar pattern was observed for HPLC compared to POC whole blood samples (Fig. 2B), with a slope of 1.12 (95% CI: 1.00 — 1.23), y-intercept of −1.37 (95% CI: −2.30 to −0.45), and correlation coefficient of r = 0.94 (95% CI: 0.91 — 0.97). When comparing POC capillary samples to POC whole blood (Fig. 2C), the slope was 1.00 (95% CI 0.95 — 1.06), with a y-intercept of −0.32 (95% CI: −0.80 — 0.15) and correlation coefficient of r = 0.95 (95% CI: 0.93 — 0.97). Additionally, 6 of the 63 samples demonstrated a bias exceeding the TEa of 6.9% across all sample types.

**Fig. 3:**
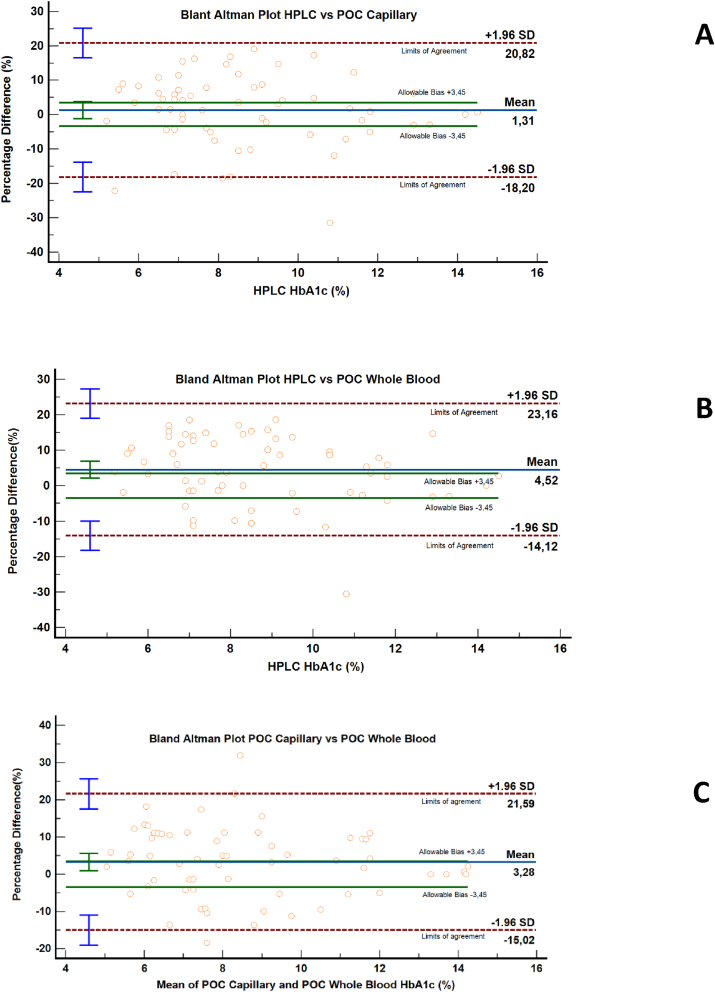
Bland Altman plot of the differences between methods (Y axis) for Finecare™ POC Capillary (A) and the Finecare ™ POC whole blood (B) compared with the Laboratory HPLC reference method (A and B, X axis) and the differences between sample types on the Finecare™ POC (C, Y axis) and mean of sample types (C, X axis). Circles indicate patients from the diabetic clinic. The green line is the allowable bias, the dashed dark red lines represent limits of agreement and the blue error bars represent 95% CI).

As illustrated in Fig. 3, the Bland Altman plots revealed a %mean difference (not % HbA1C NGSP unit) between HPLC and POC capillary of 1.3% (95% CI: −1.2 — 3.8) (Fig. 3A), between HPLC and POC whole blood of 4.5% (95% CI: 2.1 — 6.9) (Fig. 3B) and between POC capillary and POC whole blood of 3.3% (95% CI: 0.9 — 5.6) (Fig. 3C).

#### 3.2.2 Hb Variant results

**Fig. 4:**
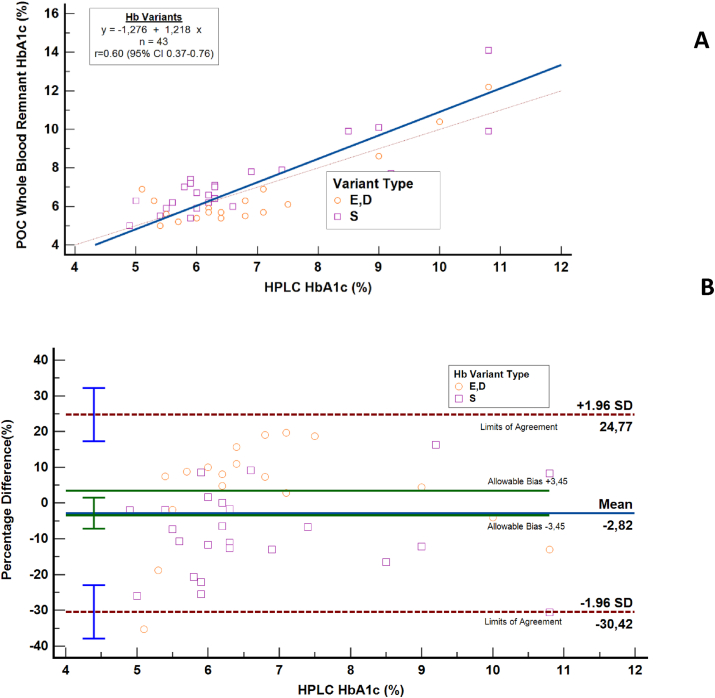
**A** Method correlation of the BIORAD Variant II HPLC Method compared to Finecare™ POC whole blood remnant samples in identified Hb Variants samples using Passing Bablok regression analysis (A). The dotted purple line is the line of identity (y = x), the solid blue line is the regression line. **B** Bland Altman plot of the differences between the methods for HPLC and POC whole blood remnant (Y axis) compared with Laboratory HPLC reference method (X-axis). The green line denotes allowable bias, the dashed dark red lines represent limits of agreement and the blue error bars represent 95% CI. Circles represent HbE, HbD and squares represent HbS.

Passing Bablok regression analysis of Hb Variant samples yielded a slope of 1.22 (95% CI: 0.94 — 1.53) with a y-intercept of −1.28 (95% CI: −3.26 — 0.45). The Spearman rank correlation coefficient between the two methods was r = 0.60 (95% CI: 0.37 — 0.77) (Fig. 4A). The Bland Altman plot revealed a %mean difference in HbA1c measurement for HPLC vs POC whole blood remnant samples in the presence of Hb Variant of −2.8% (95% CI: −7.2 — 1.5) (Fig. 4B). 31 out of 43 Hb variant samples demonstrated bias exceeding TEa.

## 4 Discussion

This study is the first to our knowledge to evaluate the performance of the Wondfo® Finecare™ FIA Meter Plus FS-113 HbA1c Rapid Quantitative Test for HbA1c measurement against a laboratory ion-exchange HPLC, which is NGSP certified and has documented traceability to the DCCT reference assay. Overall, the POC device demonstrated good agreement with the laboratory method. It is also the first study to evaluate the performance of this POC device in the presence of Hb variant samples, where poor performance was observed.

The Finecare™ POC device demonstrated good performance in measuring HbA1c using both whole blood and capillary samples, yielding results comparable to the HPLC method. However, capillary samples emerged as the preferred sample type, as the mean bias of 1.3% compared to the HPLC method fell within the derived IFCC recommendations, suggesting suitability for routine monitoring. In contrast, the mean bias of 4.5% (95% CI: 2.1 — 6.9%) observed between POC whole blood and laboratory method exceeded the derived IFCC recommendations. However, since the lower limits of the confidence interval fell within acceptable limits, the POC device's performance cannot be considered unacceptable with 95% confidence for whole blood samples. Furthermore, the correlation was poor in the presence of Hb variants, raising concerns about performance in populations with a high prevalence of Hb variants. The precision verification study findings showed that although the device's within-assay and within-laboratory CVs met the manufacturer's CV <10% specification, they exceeded both the derived IFCC CV target of <1.7% and the more attainable <3% benchmark proposed by Lenters-Werner et al. [34]. This level of imprecision emphasises the limitations of POC devices in diagnosing diabetes and aligns with clinical diabetes guidelines, which recommend that for diagnostic purposes, HbA1c should be performed in a laboratory using a NGSP-certified method standardised and traceable to the DCCT reference assay [[12], [13], [14]].

Although HbA1c POC analysis cannot currently be used for the diagnosis of DM, literature has been divided on its performance and utility for monitoring purposes. Whilst various studies worldwide have confirmed the validity of POC devices in managing known diabetic patients, and the comparability of HbA1c results obtained from POC analysis to formal laboratory methods, other studies, including a systematic review and meta-analysis of 61 studies, have highlighted limitations, with some devices showing poor performance or substantial variability in bias [21,35,36]. A study conducted in the Middle East also using the Finecare™ HbA1c POC device demonstrated good agreement when compared to the Roche Cobas Pro c503, a laboratory-based turbidimetric inhibition immunoassay, with a correlation coefficient of r > 0.9 in both capillary and whole blood samples, consistent with our study [37]. Similarly, several other studies have also reported good agreement between POC devices and laboratory-based methods such as HPLC and immunoassay [[38], [39], [40]]. Such findings support the use of POC HbA1c in monitoring, especially in known diabetic patients, where rapid turnaround time may facilitate treatment decisions. In contrast, other evaluations have reported significant limitations, with many POC devices demonstrating inaccurate or imprecise performance, and failing to meet NGSP and/or IFCC certification criteria [36,[41], [42], [43]]. These discrepancies may reflect the effect of lot-to-lot variation, issues with calibration/standardisation, differences in analytical principles, operator variability and experience, as well as matrix effect, which could also explain why bias exceeded the TEa in a small proportion of samples in Cohort A [[41], [42], [43]]. The limits of agreement were also wide across all sample types, illustrating a broad dispersion in the differences between the POC and laboratory method and highlighting potential variability in performance between the two methods. The need to participate in External Quality Assurance (EQA) testing programs for POC users has also been mandated to ensure quality [41,42]. Furthermore, it was observed that as most of these studies use rigorous evaluation protocols, POC performance may worsen when used in routine clinical practice, as opposed to ideal research settings [43]. In our study, although the Finecare™ POC device was not IFCC or NGSP certified at the time of our evaluation, the observed bias remained within acceptable analytical performance limits, supporting its potential utility in clinical monitoring of glycaemic control.

Capillary sampling, which may be influenced by multiple factors related to collection technique, surprisingly emerged as the preferred sample type in our study [44].

The Finecare™ POC instrument is designed for both capillary and whole blood samples, and while we observed a strong correlation between these two sample types, the mean bias for whole blood relative to laboratory HPLC was unexpectedly higher than that observed for capillary samples. These findings contrast with reports of minimal bias between whole blood and laboratory methods, as well as consistency between sample types on other POC platforms [37,45]. Notably, another study which also assessed the performance of the Finecare™ device, reported a small mean bias of 0.003% (%NGSP units) between whole blood and a laboratory method, and mean bias of 0.047% (% NGSP unit) between the two sample types [37]. The bias we observed using whole blood samples may be attributed to the steps involved in sample preparation where errors can possibly be introduced, especially as multiple manual pipetting steps are required for whole blood but not capillary samples. An outlier is also evident on the Bland-Altman plot for Cohort A. Outliers were retained to reflect the real-world performance of the POC device. The deviation observed may be attributable to preanalytical factors, such as sample handling, which remain an important consideration in POC testing. Further studies are needed for this POC device to determine whether the two sample types can be used interchangeably without affecting clinical outcomes.

We found poor correlation between HPLC and POC whole blood samples in the presence of Hb variants, indicating potential interference.

Although the mean bias observed was within defined performance specifications, the potential for misinterpretation remains and highlights how over-reliance on central tendency statistics can lead to erroneous conclusions. The wide confidence intervals around the mean bias, poor correlation using regression statistics and the higher proportion of samples with a bias exceeding TEa illustrate the heterogeneity introduced by Hb variants, when comparing immunoassay-based POC results to laboratory HPLC. Over 1400 hemoglobin variants have been discovered to date, many of which are clinically silent if heterozygous, potentially leading to inaccurate HbA1c results [23,46]. Such interferences are especially problematic with immunoassay-based POC devices, where they can go undetected, and may impact glycaemic management by failing to trigger appropriate therapeutic adjustments [23]. This pattern is consistent with numerous reports showing that Hb variants interfere with POC HbA1c measurement, even in devices certified by NGSP or IFCC [43]. Evidence across different POC platforms demonstrate significant susceptibility to Hb variant interference [47,48]. The consistency of these findings reinforces our observation that, whenever Hb variants are a realistic possibility, as they are across much of Africa, POC HbA1c should not be relied upon as the sole measure of glycaemic control, as undetected interferences may lead to inappropriate diabetes management.

This study has several strengths. Notably, it is the first to evaluate the Finecare™ HbA1c POC device, which uses fluorescence immunoassay, against a NGSP certified laboratory HPLC method, known to potentially identify Hb variants. This comparison is particularly important because evaluating an immunoassay-based POC device against another immunoassay-based laboratory method would not reveal limitations associated with this assay type. HPLC methodologies offer separation of Hb fractions which can highlight potential interferences due to Hb variants. By including both capillary and whole blood samples, our study also reflects real-world clinical practice. Furthermore, no prior research worldwide has examined the impact of Hb variants identified by HPLC on the accuracy of this POC device, making this an important contribution to the field. However, this study has its limitations, including a small sample size in both cohorts. It did not assess the clinical impact across different HbA1c concentrations or consider conditions affecting red blood cell lifespan, such as iron deficiency anaemia, renal failure, or Human Immunodeficiency Virus (HIV) and Tuberculosis, which are prevalent in low-to middle-income countries. This study was also conducted by a trained clinician with laboratory experience, which may not be reproducible in all settings. Additionally, Hb variants were identified based on chromatogram peaks without genetic confirmation or Hb electrophoresis. Lastly, while POC devices may perform well in evaluation studies, their effectiveness in clinical settings also depends on proper maintenance, trained personnel, and inclusion in EQA programs [49]. Further studies exploring the long-term clinical and economic impacts of integrating POC devices into routine care, with larger sample sizes and gold standard detection of potential interferences are warranted.

## 5 Conclusion

The Finecare™ HbA1c POC device demonstrated acceptable performance with both capillary and whole blood samples, comparable to the laboratory HPLC method, although capillary samples would be preferrable. Although not suitable for diagnosis, it is reliable for monitoring diabetic patients, offering advantages like short analysis times and small sample volume requirements. In settings where there is limited timely access to laboratory HbA1c, POC HbA1c testing may serve as a valuable adjunct to laboratory services and help reduce delays in clinical decision-making. However, its use as an alternative to laboratory HPLC should be approached with caution in populations with a high prevalence of Hb variants, including heterozygous carriers. It is not recommended to be used in patients with known haemoglobinopathies as these may lead to inaccurate results and incorrect clinical management. In such instances, continued use of laboratory methods or alternative methods not subject to interference is recommended. POC testing may have a role in ensuring equitable access to quality diabetes monitoring, however thorough evaluation of devices is needed to ensure they are fit-for-purpose in the intended population.

## CRediT authorship contribution statement

**Bibi Mohungoo Khan:** Conceptualization, Data curation, Formal analysis, Investigation, Methodology, Writing – original draft, Writing – review & editing. **Sean Currin:** Conceptualization, Methodology, Supervision, Writing – review & editing. **Farzahna Mohamed:** Supervision, Writing – review & editing. **Doreen Jacob:** Conceptualization, Project administration, Supervision, Writing – review & editing.

## Declaration of competing interest

The authors declare the following financial interests/personal relationships which may be considered as potential competing interests: BFS Mohungoo Khan reports equipment, drugs, or supplies was provided by IVC Health Pty LTD. If there are other authors, they declare that they have no known competing financial interests or personal relationships that could have appeared to influence the work reported in this paper.

## Data Availability

Data will be made available on request.
