# Supplementary material for: Evaluation of a point-of-care testing platform against a laboratory HbA1c measurement method in a South African tertiary-level diabetes clinic
Source: Pract Lab Med. 2026 Jul 1;51:e00547. doi: 10.1016/j.plabm.2026.e00547 (PMC13352035; doi:10.1016/j.plabm.2026.e00547)
Supplement: Multimedia component 1 [file mmc1.docx]

**Supplementary Data**

**Supplementary Table 1:** Point of care device precision study using Internal Quality Control solutions from an external provider.

IQC, internal quality control; CV, coefficient of variation, TEa, Total Allowable Error

| **IQC level** | **Assigned Range (%)** | **Assigned Mean** | **Device Mean (%)** | **Bias** | **Within Assay CV (%)** | **Within**  **Laboratory CV (%)** |
| --- | --- | --- | --- | --- | --- | --- |
| Level 1 | 4.48-6.72 | 5.6 | 5.7 | 0.1 | 2.8 | 4.0 |
| Level 2 | 8.36-12.5 | 10.5 | 11.0 | 0.5 | 2.6 | 3.0 |
